# Supplementary material for: Multidrug-Resistant and Extensively Drug-Resistant Escherichia coli in Sewage in Kuwait: Their Implications
Source: Microorganisms. 2023 Oct 23;11(10):2610. doi: 10.3390/microorganisms11102610 (PMC10609297; doi:10.3390/microorganisms11102610)
Supplement: Supplementary file 1 [file microorganisms-11-02610-s001.zip › Supplementary Table S1.pdf]

Supplementary Table S1. Antibiotics that were used with their resistance or susceptible zone diameters (mm).[a]

**Antibiotic class:** Antibiotic (amount in the disk)	Resistant	Intermediate-resistant	Susceptible
**Aminoglycosides**			
Gentamicin (10 µg)	<13	13-14	>14
Streptomycin (10 µg)	<12	12-14	>14
Amikacin (30 µg)	<15	15-16	>16
**Cephalosporins**			
Cefepime (30 µg)	<19	19-24	>24
Cephalothin (30 µg)	<15	15-17	>17
Ceftazidime (30 µg)	<18	18-20	>20
Cefoxitin (30 µg)	<15	15-17	>17
Cefotaxime (30 µg)	<23	23-25	>25
Ceftriaxone (30 µg)	<20	20-22	>22
**Penicillins with β-lactamase inhibitors**			
Amoxyclav (Amoxicillin/Clavulanic acid) [30 µg (20/10 µg)]	<14	14-17	>17
Ampicillin/Sulbactam (10/10 µg)	<12	12-14	>14
Piperacillin/Tazobactam (100/10 µg)	<18	18-20	>20
**Penicillin**			
Ampicillin (10 µg)	<14	14-16	>16
**Ureidopenicillin**			
Piperacillin (100 µg)	<18	18-20	>20
**Monobactam**			
Aztreonam (30 µg)	<18	18-20	>20
**Antifolates**			
Trimethoprim (5 µg)	<11	11-15	>15
Co-trimoxazole (Sulpha/Trimethoprim) (25 µg [23.75/1.25 µg])	<11	11-15	>15
**Tetracycline**			
Tetracycline (30 µg)	<12	12-14	>14
**Carbapenems**			
Ertapenem (10 µg)	<19	19-21	>21
Meropenem (10 µg)	<20	20-22	>22
Imipenem (10 µg)	<20	20-22	>22

**Fluoroquinolone**<br>Ciprofloxacin (5 µg)	<16	16-20	>20
**Phosphonic acid derivative**<br>Fosfomycin (200 µg)	<13	13-15	>15
**Macrolide**<br>Azithromycin (15 µg)	≤12	–	≥13
**Chloramphenicol**<br>Chloramphenicol (30 µg)	<13	13-17	>17
**Sulphonamide**<br>Sulphafurazole (Sulfisoxazole) (300 µg)	<13	13-16	>16
**Polymyxins**<br>Polymyxin B<br>Colistin	Growth in polymyxin broth[a]	NA[b]	NA[b]

[a]Bacteria were tested for susceptibiity to polymyxin B and colistin by Rapid Polymyxin NP test.
[b]Not applicable
