# Supplementary material for: Multidrug-Resistant and Extensively Drug-Resistant Escherichia coli in Sewage in Kuwait: Their Implications
Source: Microorganisms. 2023 Oct 23;11(10):2610. doi: 10.3390/microorganisms11102610 (PMC10609297; doi:10.3390/microorganisms11102610)
Supplement: Supplementary file 1 [file microorganisms-11-02610-s001.zip › Supplementary Table S2.pdf]

Supplementary Table S2. List of primers used and the cycling parameters for each PCR assay.

Gene/Clone	Specific gene	Primer sequence	PCR parameters	Product size (bp)	Reference
Antibiotic resistance genes (cephalosporinase [ESBL] genes)	*bla*$_{\text{oxa-1}}$	F: 5’- CGCAAATGGCACCAGATTCAAC-3’	94°C/ 30 s, 63.5°C/ 90 s, 72°C/ 90 s (30 cycles)	464	1
		R: 5’-TCCTGCACCAGTTTTCCCATACAG-3’			
	bla$_{\text{CMY-2}}$	F: 5’-TGATGCAGGAGCAGGCTATTCC-3’		323	
		R: 5’-CTAACGTCATCGGGGATCTGC-3’			
	bla$_{\text{ampC}}$	F: 5’-TGAGTTAGGTTCGGTCAGCA-3’		98	
		R: 5’-AGTATTTTGTTGCGGGATCG-3’			
	bla$_{\text{CTX-M}}$	F: 5’-ATGTGCAGYACCAGTAARGTKATGGC-3’		593	
		R: 5’-TGGGTRAARTARGTSACCAGAAYCAGCGG-3’			
	bla$_{\text{TEM}}$	F: 5’-TTGCTCACCCAGAAACGCTGGTG-3’		708	
		R: 5’-TACGATACGGGAGGGCTTACC-3’			
	bla$_{\text{SHV}}$	F: 5’-CGCCGGGTTATTCTTATTTGTCGC-3’		1,016	
		R: 5’-TCTTTCCGATGCCGCCGCCAGTCA-3’			
Antibiotic resistance genes (carbapenemase-encoding genes)	*bla*$_{\text{NDM}}$	F: 5’-GGTGCATGCCCGGTGAAATC-3’	94°C/ 30 s, 60°C/ 90 s, 72°C/ 60 s (30 cycles)	660	
		R: 5’-ATGCTGGCCTTGGGGAACG-3’			
	bla$_{\text{OXA-48}}$	F: 5’-TTGGTGGCATCGATTATCGG-3’		744	
		R: 5’-GAGCACTTCTTTTGTGATGGC-3’			
	bla$_{\text{KPC}}$	F: 5’-ATGTCACTGTATCGCCGTC-3’		863	
		R: 5’-AATCCCTCGAGCGCGAGT-3’			
	bla$_{\text{IMP}}$	F: 5’-CCWGATTTAAAAATYGARAAGCTTG-3’		522	
		R: 5’-TGGCCAHGCTTCWAHATTTGCRTC-3’			
	bla$_{\text{VIM}}$	F: 5’-GTTTGGTCGCATATCGCAAC-3’		382	
		R: 5’-AATGCGCAGCACCAGGATAGAA-3’			
	bla$_{\text{GES}}$	F: 5’-ATCAGCCACCTCTCAATGG-3’		302	
		R: 5’-TAGCATCGGGACACATGAC-3’			
ST131	ST131	F: 5’-GACTGCATTTCGTCGCCATA-3’	94°C/ 30 s, 60°C/ 30 s, 72°C/ 30 s (30 cycles)	310	2
		R: 5’-CCGGCGGCATCATAATGAAA-3’			
ST648	*icd96*	F: 5’-ACCACTCCGGTTGGTGGT-3’	94°C/ 20 s, 67°C/ 45 s, (30 cycles)	297	3
		R: 5’-AGAACACGGCTTAATACCGATG-3’			
	gyrB87	F: 5’-ATGGTGCGTTTCTGGCCC-3’		143	
		R: 5’-TCTTTGCCGTCGCGCTTA-3’			
	uidA	F: 5’-GCGTCTGTTGACTGGCAGGTGGTGG-3’		510	
		R: 5’-GTTGCCCGCTTCGAAACCAATGCCT-3’			

References for Table S2.

1. Fernando DM, Tun HM, Poole J, Patidar R, Li R, Mi R, et al. Detection of antibiotic resistance genes in source and drinking water samples from a First Nations community in Canada. Appl Environ Microbiol. 2016; 82: 4767–4775.

2. Doumith M, Day M, Ciesielczuk H, Hope R, Underwood A, Reynolds R, et al. Rapid identification of major *Escherichia coli* sequence types causing urinary tract and bloodstream infections. J Clin Microbiol. 2015; 53: 160–166.

3. Johnson JR, Johnston BD, Gordon DM. Rapid and specific detection of the

Escherichia coli sequence type 648 complex within phylogroup F. J Clin Microbiol. 2017; 55: 1116–1121.
