# Supplementary material for: Multidrug-Resistant and Extensively Drug-Resistant Escherichia coli in Sewage in Kuwait: Their Implications
Source: Microorganisms. 2023 Oct 23;11(10):2610. doi: 10.3390/microorganisms11102610 (PMC10609297; doi:10.3390/microorganisms11102610)
Supplement: Supplementary file 1 [file microorganisms-11-02610-s001.zip › Supplementary Table S3.pdf]

Supplementary Table S3. Distribution of resistance in ExPEC (n=14), DEC (n=3) and global clones[a] (n=4)

Antimicrobial agent	No. (%) of isolates resistant in		
	ExPEC	DEC	Global clones
Cefepime	7 (50.0)	1 (33.3)	2 (50.0)
Cephalothin	14 (100.0)	3 (100.)	4 (100.0)
Ceftazidime	8 (57.1)	2 (66.7)	2 (50.0)
Cefoxitin	5 (35.7)	2 (66.7)	3 (75.0)
Cefotaxime	14 (100.0)	3 (100.0)	4 (100.0)
Ceftriaxone	8 (57.1)	1 (33.3)	2 (50.0)
Aztreonam	7 (50.0)	1 (33.3)	3 (75.0)
Ertapenem	1 (7.1)	0 (0.0)	1 (25.0)
Meropenem	0 (0.0)	0 (0.0)	0 (0.0)
Imipenem	1 (7.1)	0 (0.0)	0 (0.0)
Gentamicin	1(7.1)	0 (0.0)	0 (0.0)
Streptomycin	8 (57.1)	1 (33.3)	4 (100.0)
Amikacin	3 (21.4)	0 (0.0)	2 (50.0)
Amoxyclav	9 (64.3)	1 (33.3)	4 (100.0)
Ampicillin/Sulbactam	4 (28.6)	1 (33.3)	1 (25.0)
Piperacillin/Tazobactam	5 (35.7)	1 (33.3)	2 (50.0)
Ampicillin	10 (71.4)	2 (66.7)	4 (100.0)
Piperacillin	13 (92.9)	1 (33.3)	4 (100.0)
Trimethoprim	5 (35.7)	1 (33.3)	1 (25.0)
Co-Trimoxazole	3 (21.4)	1 (33.3)	1 (25.0)
Tetracycline	7 (50)	1 (33.3)	3 (75.0)
Ciprofloxacin	8 (57.1)	1 (33.3)	4 (100.0)
Fosfomycin	1 (7.1)	0 (0.0)	0 (0.0)
Azithromycin	3 (21.4)	1 (33.3)	2 (50.0)
Chloramphenicol	4 (28.6)	0 (0.0)	1 (25.0)
Sulphafurazole	6 (42.9)	1 (33.3)	3 (75.0)
Polymyxin B	4 (28.6)	0 (0.0)	0 (0.0)
Colistin	2 (14.3)	0 (0.0)	0 (0.0)

[a]ST 131, ST 648
