# Supplementary material for: Multidrug-Resistant and Extensively Drug-Resistant Escherichia coli in Sewage in Kuwait: Their Implications
Source: Microorganisms. 2023 Oct 23;11(10):2610. doi: 10.3390/microorganisms11102610 (PMC10609297; doi:10.3390/microorganisms11102610)
Supplement: Supplementary file 1 [file microorganisms-11-02610-s001.zip › Supplementary Table S4.pdf]

Supplementary Table S4. Differences in resistance of CR *E. coli* (n=14) and CS *E. coli* (n=126) to other antibiotics.

Antimicrobial agent	No. of CR *E. coli*[a] (%)	No. of CS *E. coli* (%)	P-value by Chi-square test
Cefepime	13 (92.9)	58 (46.0)	0.0011
Cephalothin	14 (100.0%)	121 (96.0)	1.00
Ceftazidime	10 (71.4)	59 (46.8)	0.10
Cefoxitin	6 (42.9)	27 (21.4)	0.10
Cefotaxime	13 (92.9)	109 (86.5)	0.69
Ceftriaxone	5 (35.7)	40 (31.8)	0.76
Aztreonam	6 (42.9)	37 (29.4)	0.36
Gentamicin	0 (0.0)	7 (5.6)	1.00
Streptomycin	11 (78.6)	69 (54.8)	0.15
Amikacin	4 (28.6)	17 (13.5)	0.24
Amoxyclav	10 (71.4)	74 (58.7)	0.41
Ampicillin/Sulbactam	9 (64.3)	41 (32.5)	0.040
Piperacillin/Tazobactam	8 (57.1)	67 (53.2)	1.00
Ampicillin	13 (92.9)	80 (63.5)	0.040
Piperacillin	11 (78.6)	91 (72.2)	0.76
Trimethoprim	12 (85.7)	31 (24.6)	0.0001
Co-Trimoxazole	10 (71.4)	32 (25.4)	0.0009
Tetracycline	11 (78.6)	54 (42.9)	0.021
Ciprofloxacin	10 (71.4)	38 (30.2)	0.0052
Fosfomycin	3 (21.4)	3 (2.4)	0.014
Azithromycin	3 (21.4)	11(8.7)	0.15
Chloramphenicol		20 (15.9)	0.0002

	9 (64.3)		
Sulphafurazole	9 (64.3)	59 (46.8)	0.27
Polymyxin B	3 (21.4%)	22 (17.5%)	0.72
Colistin	2 (14.3%)	2 (1.6%)	0.05

Resistant and intermediate-resistant isolates were merged.
