# Supplementary material for: Multidrug-Resistant and Extensively Drug-Resistant Escherichia coli in Sewage in Kuwait: Their Implications
Source: Microorganisms. 2023 Oct 23;11(10):2610. doi: 10.3390/microorganisms11102610 (PMC10609297; doi:10.3390/microorganisms11102610)
Supplement: Supplementary file 1 [file microorganisms-11-02610-s001.zip › Supplementary Table S5.pdf]

**Supplementary Table S5.** Differences in resistance of ExPEC (n=14) versus non-ExPEC (n=126) isolates.

| Antimicrobial agent | No. (%) of isolates resistant in | | P-value by Chi-square test |
|---|---|---|---|
| | ExPEC | Non-ExPEC | |
| Cefepime | 7 (50.0) | 63 (50.0) | 1.00 |
| Cephalothin | 14 (100.0) | 117 (92.6) | 0.600 |
| Ceftazidime | 8 (57.1) | 59(46.8) | 0.58 |
| Cefoxitin | 5 (35.7) | 25 (19.8) | 0.18 |
| Cefotaxime | 14 (100.0) | 105 (83.3) | 0.13 |
| Ceftriaxone | 8 (57.1) | 34 (27.0) | 0.03 |
| Aztreonam | 7 (50.0) | 34 (27.0) | 0.12 |
| Ertapenem | 1 (7.1) | 3 (2.4) | 0.31 |
| Meropenem | 0 (0.0) | 2 (1.6) | 1.00 |
| Imipenem | 1 (7.1) | 10 (7.9) | 1.00 |
| Gentamicin | 1(7.1) | 6 (4.8) | 0.53 |
| Streptomycin | 8 (57.1) | 70 (55.6) | 1.00 |
| Amikacin | 3 (21.4) | 17 (13.5) | 0.42 |
| Amoxyclav | 9 (64.3) | 72 (57.1) | 1.00 |
| Ampicillin/Sulbactam | 4 (28.6) | 46 (36.5) | 0.57 |
| Piperacillin/Tazobactam | 5 (35.7) | 70 (55.6) | 0.17 |
| Ampicillin | 10 (71.4) | 97 (77.0) | 0.74 |
| Piperacillin | 13 (92.9) | 105 (83.3) | 0.70 |
| Trimethoprim | 5 (35.7) | 55 (43.7) | 0.78 |
| Co-Trimoxazole | 3 (21.4) | 55 (43.7) | 0.15 |
| Tetracycline | 7 (50) | 74 (58.7) | 0.58 |
| Ciprofloxacin | 8 (57.1) | 38 (30.2) | 0.07 |
| Fosfomycin | 1 (7.1) | 3 (2.4) | 0.35 |
| Azithromycin | 3 (21.4) | 10 (7.9) | 0.13 |
| Chloramphenicol | 4 (28.6) | 23 (18.3) | 0.47 |
| Sulphafurazole | 6 (42.9) | 60 (47.6) | 0.79 |
| Polymyxin B | 4 (28.6) | 21 (16.7) | 0.28 |
| Colistin | 2 (14.3) | 2 (1.6) | 0.05 |
