# Supplementary material for: Multidrug-Resistant and Extensively Drug-Resistant Escherichia coli in Sewage in Kuwait: Their Implications
Source: Microorganisms. 2023 Oct 23;11(10):2610. doi: 10.3390/microorganisms11102610 (PMC10609297; doi:10.3390/microorganisms11102610)
Supplement: Supplementary file 1 [file microorganisms-11-02610-s001.zip › Supplementary Table S6.pdf]

Supplementary Table S6. Cephalosporin resistance, ESBL genes, and production of ESBL.

Sample no.	Resistance pattern	Presence of resistance gene(s)	Production of ESBL[a]
J133, J211	R*: Cefepime + Cephalothin + Ceftazidime + Cefoxitin + Cefotaxime + Ceftriaxone	J133: $bla_{\text{OXA-1}}$ + *ampC* + $bla_{\text{CTX-M}}$ + bla_{TEM}	+
		J211: $bla_{\text{OXA-1}}$ + $bla_{\text{CMY-2}}$ + *ampC*	+
Z304	IR**: Cefepime + Cefoxitin<br>R: Cephalothin + Ceftazidime + Cefotaxime + Ceftriaxone	*ampC* + $bla_{\text{CTX-M}}$ + bla_{TEM}	+
Z92, H164	IR: Cefepime<br>R: Cephalothin + Ceftazidime + Cefoxitin + Cefotaxime + Ceftriaxone	Z92: $bla_{\text{CMY-2}}$ + *ampC*	-
		H164: $bla_{\text{CMY-2}}$ + *ampC* + bla_{TEM}	-
Z25, J293	IR: Cefoxitin<br>R: Cefepime + Cephalothin + Ceftazidime + Cefotaxime + Ceftriaxone	Z25: *ampC* + $bla_{\text{CTX-M}}$ + bla_{TEM}	-
		J293: *ampC* + $bla_{\text{CTX-M}}$	+
Z138	R: Cefepime + Cephalothin + Ceftazidime + Cefoxitin + Ceftriaxone	*ampC* + bla_{TEM}	-
H58	IR: Ceftazidime + Ceftriaxone<br>R: Cephalothin + Cefoxitin + Cefotaxime	$bla_{\text{CMY-2}}$ + *ampC*	-
Z277	IR: Ceftriaxone<br>R: Cephalothin + Ceftazidime + Cefoxitin + Cefotaxime	*ampC*	-
J76, H126, J181	IR: Cefepime + Ceftazidime + Cefoxitin<br>R: Cephalothin + Cefotaxime	J76: *ampC*	-
		H126, J181: *ampC* + bla_{TEM}	
J152	IR: Cefepime + Cephalothin + Ceftazidime + Cefoxitin<br>R: Cefotaxime	*ampC*	-
H80	IR: Cefepime + Ceftazidime<br>R: Cephalothin + Cefoxitin + Cefotaxime	$bla_{\text{OXA-1}}$ + *ampC*	-
H29	IR: Cefepime + Cefoxitin<br>R: Cephalothin + Ceftazidime + Cefotaxime	*ampC* + bla_{TEM}	-
H300	IR: Cefoxitin<br>R: Cefepime + Cephalothin + Ceftazidime + Cefotaxime	*ampC*	-
H248	IR: Ceftriaxone<br>R: Cefepime + Cephalothin + Cefoxitin + Cefotaxime	*ampC*	-
J154	IR: Cefepime + Cephalothin + Ceftazidime + Ceftriaxone<br>R: Cefotaxime	*ampC*	-
J22, J183	IR: Cefepime + Ceftazidime<br>R: Cephalothin + Cefotaxime + Ceftriaxone	J22: *ampC* + $bla_{\text{CTX-M}}$ + bla_{TEM}	-
		J183: *ampC* + bla_{TEM}	
J132, H306	IR: Cefepime + Ceftazidime + Ceftriaxone<br>R: Cephalothin + Cefotaxime	*ampC* + bla_{TEM}	-

J2, H75, H99, J185, Z199, H189, H224, H225, Z252, J264, Z275, Z302, Z303	R: Cefepime + Cephalothin + Ceftazidime + Cefotaxime + Ceftriaxone	J2: bla_{OXA-1} + *ampC* + bla_{TEM}	J2, H75, H99, J185, Z199, H224, H225, Z252, J264, Z275, Z302, Z303: +
		H75: *ampC* + bla_{CTX-M}	H189: -
		H99, J185: bla_{OXA-1} + *ampC* + bla_{CTX-M}	
		Z199, H189, H224, H225, J264, Z75: *ampC* + bla_{CTX-M}	
		Z252, Z302, Z303: *ampC* + bla_{CTX-M} + bla_{TEM}	
J236	IR: Cefepime + Cephalothin + Cefotaxime + Ceftriaxone	bla_{CMY-2} + *ampC*	-
Z225, Z227	IR: Cefepime + Ceftriaxone<br>R: Cephalothin + Cefotaxime	*ampC* + bla_{TEM}	-
J262	IR: Cephalothin + Ceftazidime + Ceftriaxone<br>R: Cefotaxime	*ampC*	-
Z248	IR: Ceftazidime + Cefotaxime<br>R: Cephalothin + Ceftriaxone	*ampC*	-
J207	IR: Ceftazidime + Ceftriaxone<br>R: Cephalothin + Cefotaxime	*ampC*	-
J295	IR: Cefoxitin<br>R: Cephalothin + Cefotaxime + Ceftriaxone	*ampC*	-
J291	IR: Cefoxitin + Cefotaxime<br>R: Cephalothin + Ceftriaxone	bla_{CMY-2} + *ampC*	-
J45, Z220	IR: Cefoxitin + Cefotaxime + Ceftriaxone<br>R: Cephalothin	J45: *ampC*	-
		Z220: bla_{OXA-1} + bla_{CMY-2} + *ampC* + bla_{TEM}	
H24, H112, J158	IR: Cefepime + Cefoxitin<br>R: Cephalothin + Cefotaxime	H24, H112: *ampC*	-
		J158: *ampC* + bla_{TEM}	
J28	IR: Cephalothin + Ceftazidime + Cefoxitin<br>R: Cefotaxime	*ampC*	-
Z307	IR: Cefoxitin<br>R: Cephalothin + Ceftazidime + Cefotaxime	*ampC*	-
H97	IR: Cefepime + Cephalothin + Ceftazidime + Cefotaxime	*ampC* + bla_{TEM}	-
J296	IR: Cefepime<br>R: Cephalothin + Ceftazidime + Cefotaxime	*ampC*	-
	IR: Cefepime + Ceftazidime + Cefotaxime	J209, H305: *ampC* + bla_{TEM}	-

J209, J235, H305	R: Cephalothin	J235: *ampC*	
J25, H31, J135, J260, H293	IR: Cefepime + Cephalothin + Ceftazidime<br>R: Cefotaxime	J25, J135, J260: *ampC*	-
		H31: *ampC* + *bla*$_{\text{TEM}}$	
		H293: *bla*$_{\text{OXA-1}}$ + *ampC*	
H122, J153	IR: Cefepime + Ceftazidime<br>R: Cephalothin + Cefotaxime	H122: *ampC* + *bla*$_{\text{TEM}}$	-
		J153: *ampC*	
Z60, J79	IR: Ceftazidime<br>R: Cefepime + Cephalothin + Cefotaxime	Z60: *ampC* + *bla*$_{\text{CTX-M}}$	Z60: +
		J79: *ampC* + *bla*$_{\text{CTX-M}}$ + *bla*$_{\text{TEM}}$	J79: -
J24, Z222	R: Cefepime + Cephalothin + Ceftazidime + Cefotaxime	J24: *ampC* + *bla*$_{\text{TEM}}$	J24: -
		Z222: *ampC* + *bla*$_{\text{CTX-M}}$	Z222: +
J294	IR: Cefepime + Ceftazidime<br>R: Cephalothin	*ampC* + *bla*$_{\text{TEM}}$	-
H192	IR: Ceftazidime + Ceftriaxone<br>R: Cephalothin	*ampC* + *bla*$_{\text{TEM}}$	-
Z306	R: Cephalothin + Cefotaxime + Ceftriaxone	*ampC*	-
J104	IR: Cephalothin + Cefotaxime<br>R: Cefoxitin	*ampC*	-
Z201	IR: Cefoxitin + Cefotaxime<br>R: Cephalothin	*ampC* + *bla*$_{\text{CTX-M}}$ + *bla*$_{\text{TEM}}$	-
H250	R: Cephalothin + Cefoxitin + Cefotaxime	*ampC*	-
Z319	IR: Cephalothin + Ceftazidime + Cefotaxime	*ampC*	-
H124, Z281, H304	IR: Cephalothin + Ceftazidime<br>R: Cefotaxime	H124, H304: *ampC* + *bla*$_{\text{TEM}}$	-
		Z281: *ampC*	
H166	IR: Cefepime + Cephalothin + Cefotaxime	*ampC*	-
Z198	IR: Ceftazidime<br>R: Cephalothin + Cefotaxime	*ampC* + *bla*$_{\text{TEM}}$	-
H265	R: Cephalothin + Ceftazidime + Cefotaxime	*ampC*	-
H295	IR: Ceftazidime + Cefotaxime<br>R: Cephalothin	*ampC* + *bla*$_{\text{TEM}}$	-
Z169, J208, J292	IR: Cefepime + Cefotaxime<br>R: Cephalothin	Z169: *bla*$_{\text{OXA-1}}$ + *ampC*	-
		J208: *ampC* + *bla*$_{\text{TEM}}$	
		J292: *ampC*	
H109	IR: Cefepime + Cephalothin + Cefotaxime	*bla*$_{\text{CMY-2}}$ + *ampC* + *bla*$_{\text{TEM}}$	-
H103, H104	IR: Cefepime + Cephalothin<br>R: Cefotaxime	H103: *ampC*	-
		H104: *ampC* + *bla*$_{\text{TEM}}$	
J85	IR: Cefepime + Cefotaxime<br>R: Cephalothin	*ampC* + *bla*$_{\text{TEM}}$	-
H32, Z118	IR: Cefepime<br>R: Cephalothin + Cefotaxime	*ampC*	-
Z19	IR: Cefepime + Ceftazidime<br>R: Cefotaxime	*ampC*	-

Z56	IR: Cefepime<br>R: Cephalothin	*ampC*	-
H195	IR: Ceftriaxone<br>R: Cephalothin	*ampC* + *bla*$_{TEM}$	-
H168	IR: Ceftazidime<br>R: Cephalothin	*ampC*	-
H100	IR: Cefepime	*ampC*	-
Z4, H51, H78, H79, J97, H106, H110, Z226, Z278, Z280	IR: Cephalothin + Cefotaxime	Z4, J97, H106, H110, Z278: *ampC* + *bla*$_{TEM}$<br>H51, H78, H79, Z226, Z280: *ampC*	-
J232, Z305, H303	IR: Cephalothin<br>R: Cefotaxime	J232: *ampC*<br>Z305: *ampC* + *bla*$_{TEM}$<br>H303: *ampC* + *bla*$_{CTX\text{-}M}$	-
Z82, Z84, Z89, Z93, H135, H220, H222, J239, Z279, H272, H302	IR: Cefotaxime<br>R: Cephalothin	Z82, Z84, Z89, Z93, H220, J239, H272: *ampC*<br>H135, H222, Z279, H302: *ampC* + *bla*$_{TEM}$	-
Z202, J289, H301	R: Cephalothin + Cefotaxime	*ampC*	-
H159, H82	IR: Cefotaxime	*ampC*	-
H267	R: Cefotaxime	*ampC*	-
H28, J86, H77, H297, Z58, Z88	IR: Cephalothin	H28, J86, H77, H297: *ampC*<br>Z58, Z88: *ampC* + *bla*$_{TEM}$	-
J18, J23, H221, H70, J126	R: Cephalothin	J18, J23, H221: *ampC* + *bla*$_{TEM}$<br>H70, J126: *ampC*	-

[a]Extended-spectrum β-lactamase

R*: Resistant

IR**: Intermediate resistant
