# Supplementary material for: Multidrug-Resistant and Extensively Drug-Resistant Escherichia coli in Sewage in Kuwait: Their Implications
Source: Microorganisms. 2023 Oct 23;11(10):2610. doi: 10.3390/microorganisms11102610 (PMC10609297; doi:10.3390/microorganisms11102610)
Supplement: Supplementary file 1 [file microorganisms-11-02610-s001.zip › Supplementary Table S7.pdf]

Supplementary Table S7. Resistance patterns of, and ESBL production by, global clones.

Sample no.	Global clones	Resistance pattern in disk diffusion test	Positive for resistance genes	ESBL production with ESBL NDP test
Z222	ST131	cefepime, cephalothin, ceftazidime, cefotaxime, aztreonam, streptomycin, amoxyclav, ampicillin, piperacillin, tetracycline, ciprofloxacin, azithromycin, sulphafurazole, polymyxin-B	*ampC*, $bla_{CTX\text{-}M}$	+
J211	ST648	cefepime, cephalothin, ceftazidime, cefoxitin, cefotaxime, ceftriaxone, aztreonam, ertapenem, streptomycin, amikacin, amoxyclav, ampicillin/sulbactam, piperacillin/tazobactam, ampicillin, piperacillin, trimethoprim, co-trimoxazole, tetracycline, azithromycin, chloramphenicol, ciprofloxacin, sulphafurazole	$bla_{OXA\text{-}1}$, $bla_{CMY\text{-}2}$, *ampC*	+
H250	ST648	cephalothin, cefoxitin, cefotaxime, streptomycin, amoxyclav, ampicillin, piperacillin, ciprofloxacin	*ampC*	-
J295	ST648	cephalothin, cefoxitin, cefotaxime, ceftriaxone, aztreonam, imipenem, streptomycin, amikacin, amoxyclav, piperacillin/tazobactam, ampicillin, piperacillin, tetracycline, ciprofloxacin, sulphafurazole	*ampC*	-
